# New Drugs, Appliances, and Things Medical

**Published:** 1891-07-25

**Authors:** 


### NEW DRUGS, APPLIANCES, AND THINGS MEDICAL.

[All preparations, appliances, novelties, etc., of which a notice is desired, should be sent for The Editor, to care of The Manager, 140, Strand, London, W.C.]

### COOMB'S "EUREKA" AERATED PASTRY FLOUR.

STANDFORD STREET, NOTTINGHAM.

The company producing the above have submitted samples of their manufacture for notice. We find that the flour is of excellent quality, and particularly well suited for the making of light and digestible pastry. In pursuance of our usual custom we have had, in addition to our own investigation, various dishes made with the flour. The results are excellent, both to the eye and taste, and our cook states that the flour is first-rate to work with. When we add that two gold medals and commendations were gained last year for the best pastry flour, and that the sale in 1890 was more than a million and a-half pounds, we think we have placed sufficient evidence before our readers of the value of this cereal food.

